# Incorporating Aboriginal women’s voices in improving care and reducing risk for women with diabetes in pregnancy - A phenomenological study

**DOI:** 10.1186/s12884-021-04055-2

**Published:** 2021-09-16

**Authors:** Anna J Wood, Sian Graham, Jacqueline A Boyle, Beverley Marcusson-Rababi, Shonada Anderson, Christine Connors, Harold D McIntyre, Louise Maple-Brown, Renae Kirkham

**Affiliations:** 1 Menzies School of Health Research, Charles Darwin University, NT Darwin, Australia; 2 Aboriginal and Torres Strait Islander Advisory Group, Menzies School of Health Research, Charles Darwin University, NT Darwin, Australia; 3 Monash Centre for Health Research and Implementation, Monash University, Vic Melbourne, Australia; 4 Top End Health Service, Northern Territory Department of Health, NT Darwin, Australia; 5 Mater Research, The University of Queensland, QLD South Brisbane, Australia; 6 Department of Endocrinology, Royal Darwin Hospital, NT 0810 Darwin, Australia

**Keywords:** Gestational Diabetes, Aboriginal and Torres Strait Islander Peoples, Pregnancy, Preventative medicine

## Abstract

**Background:**

There is a high burden of gestational diabetes (GDM) and type 2 diabetes in pregnancy for Aboriginal and Torres Strait Islander women. Postpartum diabetes programs have the potential to prevent recurrent GDM and improve management of type 2 diabetes. However, data on such programs are limited, particularly in the Indigenous context. We aimed to explore Aboriginal Australian women’s and health providers’ preferences for a program to prevent and improve diabetes after pregnancy.

**Methods:**

A phenomenological methodology underpinned semi-structured in-depth interviews with eleven Aboriginal women and seven health professionals across the Northern Territory from October 2019- February 2020. Interviews were analysed using an inductive analysis framework to address the barriers and enablers of proposed diabetes prevention programs identified by participants.

**Results:**

Identified structural barriers to lifestyle change included: food insecurity, persuasive marketing of unhealthy food options, lack of facilities and cultural inappropriateness of previous programs. Enablers to lifestyle change included: a strong link between a healthy lifestyle and connection with Country, family and community. Suggested strategies to improve lifestyle included: co-designed cooking classes or a community kitchen, team sports and structural change (targeting the social determinants of health). Lifestyle change was preferred over metformin to prevent and manage diabetes after pregnancy by participants and health care providers.

**Conclusions:**

We recommend individual level programs be designed alongside policies that address systemic inequalities. A postpartum lifestyle program should be co-designed with community members and grounded in Aboriginal conceptions of health to adequality address the health disparities experienced by Aboriginal people in remote communities.

**Supplementary Information:**

The online version contains supplementary material available at 10.1186/s12884-021-04055-2.

## Background

Rates of hyperglycaemia in pregnancy – due to pre-existing type 2 diabetes or gestational diabetes (GDM) – are disproportionately higher among Indigenous women worldwide [1, 2]. In the Australian context, Aboriginal women are 1.2 times more likely to have GDM and greater than 10 times more likely to have pre-existing type 2 diabetes than non-Indigenous women [3]. Recurrence of GDM is reported to occur in 30–84 % of subsequent pregnancies [4] and women with GDM have a sevenfold higher risk of developing type 2 diabetes compared to women without GDM [5]. The risk is higher for Aboriginal women who have a greater than fourfold risk of developing type 2 diabetes after GDM, compared with non-Indigenous women [6].

Evidence suggests that type 2 diabetes can be prevented with lifestyle and pharmacological methods [7], although the evidence in the post GDM population is not as clear [8, 9]. Trials showing type 2 diabetes can be prevented with lifestyle change, such as the Diabetes Prevention Program, generally involve expensive and time-consuming interventions [10]. This presents a challenge for mothers with young families, who commonly cite tiredness, and competing work and carer duties as barriers to lifestyle change in the postpartum period [11, 12]. To date, this evidence is primarily based on research of non-Indigenous women in urban centres and may not be applicable in other contexts. Whilst there are several lifestyle programs targeted at Indigenous populations [13, 14], there is currently no literature on specific postpartum programs in Indigenous populations worldwide.

In order to be successful, lifestyle programs need to be context specific and adaptable, ensuring they are culturally appropriate [15]. Aboriginal Australians are culturally and linguistically diverse peoples who now comprise 3 % of the Australian population. In the Northern Territory (NT), 30 % of women identify as Aboriginal and/or Torres Strait Islander and 80 % of those women live in remote communities [16]. Aboriginal women in remote Australia, experience high levels of social disadvantage, including inadequate housing, food insecurity [17] and inadequate places to exercise, serving as barriers to engagement in lifestyle change programs. Conversely, Aboriginal cultures have strong connections to ‘Country’ (a term that refers to traditional lands [18]) a potential enabler to participation in lifestyle programs.

In light of perceived barriers to lifestyle programs, pharmacological interventions have been evaluated. Metformin has been shown to prevent type 2 diabetes in the general population [19], and to be more effective at preventing type 2 diabetes in women who had GDM compared to parous women without a history of GDM [20, 21]. However, there are concerns around adherence to metformin given its gastrointestinal side effects, and the large size of the tablet [22]. Whether metformin used for primary prevention in the Aboriginal Australian context would be an acceptable and effective strategy is currently unknown.

Another important consideration for lifestyle interventions is that concepts of health for Aboriginal populations differ from the Western biomedical paradigm that drives mainstream healthcare delivery. Programs need to privilege Aboriginal people’s voices and consider the different ways cultures conceptualise health [23]. Furthermore, individual level programs are unlikely to be successful without addressing the underlying societal and economic drivers at play [24, 25]. We aimed to explore Aboriginal women’s and health providers’ preferences for a program to prevent and improve diabetes after pregnancy.

## Methods

This qualitative study was underpinned by a phenomenological methodology [26]. Interviews were conducted, with a focus on how perceptions of a diabetes prevention program were shaped by the experience of diabetes in pregnancy. It also aimed to empower and incorporate Aboriginal voices and cultural values within this research [27, 28]. This study sits within The Diabetes across the Lifecourse: Northern Australia Partnership, a partnership between researchers, policy makers and health service providers. Strategic advice concerning aspects of the Partnership is provided by an Aboriginal and Torres Strait Islander AdvisoryGroup (ATSIA), including Aboriginal women across the NT and represented here by SG, chair of the ATSIAG. The design of this study was in response to a priority set by the ATSIAG to prevent type 2 diabetes after GDM. Prior to study commencement, the ATSIAG. emphasised the importance of employing an Aboriginal Community Worker and returning to communities to ensure outcomes from this project were communicated back to those involved. In each community an Aboriginal Community Worker was recruited through established relationships between the Partnership and the communities. Whilst they had no formal research training, both women had experience in cross-cultural communication and fluency in both English and local languages. They were employed by the research team to establish a culturally safe space and ensure the appropriate interpretation of Aboriginal women’s voices.

The research team comprised of Aboriginal and non-Indigenous people, vital to establishing rapport, collecting meaningful data and making accurate interpretations of data. AW and BMR were responsible for conducting interviews and AW and SG were responsible for analysing the data. AW is an endocrinologist and PhD candidate of European ancestry. The potential power imbalance from her position was, at least in part, mitigated by interviewing in partnership with an Aboriginal community worker. BMR is an Aboriginal Wiradjuri woman with a masters by research and interviewer experience. SG is an Aboriginal Noongar and Bardi-Jawi woman, with a certificate IV in Indigenous Research Capacity Building and a Bachelor of Applied Science. RK is of European ancestry, and an early career researcher with expertise in qualitative methodologies and health systems research. RK led supervision of data collection, analysis and interpretation.

### Study setting

Participating communities responded to an initiative requesting communities to undertake this study with members of their staff and clients from Top End Health Service Remote Primary Health Care clinics in the NT. These communities (A and B) are situated in the NT and classified as very remote and socio-economically disadvantaged [29]. Local Aboriginal languages are spoken in each community with English being the 3rd or 4th language spoken.

### Study participants

The study population were Aboriginal women with a history of GDM or type 2 diabetes in pregnancy in the last five years, aged > 18 years and purposefully sampled based on these criteria. Health professionals at the remote clinics identified potential participants and the Aboriginal Community Worker in each community informed potential participants of the study and assisted with gaining consent. Health professionals were recruited through direct contact with remote clinics. A group of community advocates who self-identified as community elders were also interviewed (4 women together). This study was approved by the Human Research Ethics Committee of the NT Department of Health and Menzies School of Health Research (HREC 19-3362).

### Data collection

The content of the interview questions was developed and informed from a literature review and input from the research team, then discussed with the ATSIAG and the Aboriginal Community Workers, with no concerns raised. The interviews focussed on participants’ opinions on lifestyle changes and metformin for primary prevention of diabetes. The interview guide was piloted with initial participants (included in data analysis) and modified over the study to ensure content relevant to the aims of the study was captured and framed in an appropriate way. No further interviews were conducted once consistent findings emerged from interviews.

AW and BMR conducted interviews in community A in the presence of the Aboriginal Community Worker and AW conducted interviews in community B in the presence of an Aboriginal Community Worker from October 2019 -February 2020. Interviews were conducted in English and questions and responses in local language were translated in English by the Aboriginal Community Worker when needed. Interviews occurred at a location deemed appropriate by the participant and Community Worker (health clinic, participants’ homes or outside in a private space). Interview duration ranged from 20 to 60 min and field notes were taken by AW.

### Data analysis

Interviews were audio-recorded and transcribed verbatim by an independent transcription company. Immediately after each interview (with Aboriginal women), interpretation of meaning was discussed between AW and the Aboriginal Community Worker to ensure AW correctly interpreted women’s data and field notes were taken by AW. Participants were invited to review their transcripts, though none did.

The program NVivo (version 10) was used to guide analysis. AW and SG independently undertook the initial round of coding -analysing both transcripts and field notes- using an inductive analysis framework [30]. This involved line by line coding whereby clusters of meaning informed the development of categories highlighting participants' descriptions of experiences. Evolving themes were identified and negotiated between AW and SG until a final coding structure was determined. To cross-check the accuracy of interpretation, after the initial rounds of coding, it was planned that the Aboriginal Community Worker in each community would review themes developed. However, due to the COVID-19 pandemic, remote communities were closed for non-essential visitors in 2020 and AW was unable to revise themes in person with the Aboriginal Community Workers. Instead, AW discussed key themes with the community worker from community B via telephone, although was unable to contact the Aboriginal Community Worker in community A. Additionally, key themes were discussed with the ATSIA for advice, feedback and guidance. Recommendations from the study will be shared in feedback sessions with communities once COVID-19 pandemic restrictions are lifted.

## Results

The study sample included 11 women with a history of hyperglycaemia in pregnancy, 7 health professionals and 1 group of community advocates (4 women spoke together) (Table 1). Most individuals who were approached consented to participate (11/12 women community A, 3/5 women community B, 7/7 health professionals), with those who declined not specifying a reason for this. Two women from community A were approached and consented for follow-up interviews to further expand on previous discussions. The types of health professionals interviewed were selected as typical of those who provide healthcare for women with diabetes in remote communities.

**Table 1:** Characteristics of participants

	Women with history of diabetes in pregnancy *n* = 11	Health Professionals *n* = 7
Gestational diabetes	9 (82 %)	-
Type 2 diabetes	2 (18 %)	-
Mean Age (standard deviation)	28 (5.7)	-
Median time since birth [interquartile range]	2 years [5 months to 4 years]	-
Community A	8 (73 %)	3 (43 %)
Community B	3 (27 %)	4 (57 %)
Remote general practitioner	-	4 (57 %)
Midwife	-	2 (29 %)
Chronic disease nurse	-	1 (14 %)

Analysis of the interviews revealed themes outlined in Table 2. Whilst community A and B are unique in terms of traditional owners and language groups, themes were consistent across the communities. Quotes from women with a history of hyperglycaemia in pregnancy are labelled W1-11, quotes from health professionals are labelled HP1-7 and quotes from the community advocate group labelled C1.

**Table 2:** Summary of themes developed among participants

Main theme	Subtheme	Sample quotes
A. **Structural barriers to lifestyle change**:		
1. Food insecurity and availability	• Availability (junk food more available) • Access (enough money, other people eating food) • Utilisation (overcrowding, limited ◊ facilities, fridges, easier to buy fast-food) • Stability (weather dependent) • Sugary drinks and food are addictive • Harder to eat well	**Availability** • W11: can’t keep eating good food, sometimes we get low of veggies and stuff…Yes sometimes when we get money, we get veggies… just eat what we got in the house, like plain noodles or just sandwich, butter and bread. **Access** • HP5: women try and buy healthy foods, put it in the cupboard, it’s eaten, and it’s gone. So, you basically eat for now. So, take away food is easy. I get my meal now, I eat it, it’s mine. Whereas if you buy for the future, there’s no guarantee you’re going to eat it. **Utilisation** • W1: if you’ve got three or four different families living in one house, what are you …? That other family’s going to go in and get your food while you’re not there, unless you’ve got a lock on your fridge, and most people don’t have fridges in their house. **Stability** • HP4: keeping food fresh and also by the time it’s travelled out here, it’s already three or four days old and frozen food would be better because the nutrient balance is probably better, but they don’t have freezers to freeze their food. **Other** • HP7: if you’re under such a barrage of sales I think the actual resisting it, especially in poorer areas is much more difficult. • C1: quick feed, I’m hungry, I’ll just buy anything, Yeah, they just grab anything in the shop they will eat
2. Cultural appropriateness of facilities and previous programs	• Previous failed programs • Western concepts of exercise	• W2: it’s too hot to exercise here. • W10: the basketball court they closed it so some of them are not going there. • W1: [people jogging and swimming] They’re only the white people. You never see an Aboriginal person jogging. • W1: they used to have yoga, years ago, but it was only the nurses and the teachers that used to turn up; no locals. **What sort of exercise might work?** • W6: just women. And make it strict.
3. Competing priorities	• Other life stressors take priority • Not motivated	**Related to cooking and diet** • HP1: I think the depression and the despair is getting worse and worse and this is probably one of the worst communities I’ve seen: the hopelessness is really quite overwhelming here. **Related to sport and exercise** • W5: they got other problems, or they not interested.
B. **Enablers to lifestyle change**:		
1. Culture and connection with Country	• Going on Country • Eating bush-tucker • Look after each other’s children	• W2: most people love their bush food. I know I do. • W8: because Mum always … take me hunting and make me eat healthy food. She always cook … and make me food. • HP1: I think it needs to get back into the community. This is something that needs to come from the community **Would it be hard to exercise because you have to look after your kids?** • W2: no. They can come with me
C. **Solutions**		
1. Structural changes	• Banning soft drink • Subsidising healthy food	• W1: stop selling sugary stuff in the shop. • HP2: maybe if you ban soft drinks, I don’t know. But again, even as I say that I know you’ve got to make the decision yourself. **What do you think if you made junk food and soft drink expensive/ subsided fruit and vegetable?** • W4: they’ll keep it. They don’t care if it’s expensive they still buy it.
2. Cooking classes and community kitchen	• Culturally appropriate	• W6: to teach our people to cook, to better cooking healthy food. Need to have healthy food. • W7: maybe they’ll get all the womens together and sit and talk about what sort of food that we want to, you know, eat, some better food. And bush tuckers.

### Structural barriers to lifestyle change

#### Food insecurity and availability

Most women, and all health professionals referenced food insecurity as a key barrier to improved diet. The inability to consistently access food due to not enough money was described by W4, “*If I have enough money, I can buy groceries, good food. But sometimes when I ran out of – so I just go out to take away*”. The ability to prepare, cook and store food - due to overcrowded living conditions, limited cooking facilities and access to refrigeration - were also significant issues brought up by many, as reported by C1: “*[if you] live in a crowded house and you buy food, and then everybody else eats it.”* Problems with availability and stability of healthy food was described by one health professional:

“*Our fresh produce in the wet season is awful. You buy it and it’s already wilted and you’ve got a day to eat it and that’s it, because it comes in on a plane. There is no barge here and there is no road access for 6 months of the year*” (HP5).

Women acknowledged that it was hard to eat well, identifying the ease of takeaway foods as a key reason for choosing them: “*I couldn’t be bothered cooking when you’re working and you’re tired*” (W1). Many suggested the addictiveness and appeal of soft drinks as reasons for their widespread use, as W2 notes: *“I think Coke is just addictive, you know… I think Coke is the best friend for everybody”.*

#### Cultural appropriateness of facilities and programs

Lack of available facilities to assist with overcoming the hot climate were often stated as barriers to partaking in physical activity. It was argued that there were either “*no places to exercise*” (W2), or if there were, they were often not maintained or open to the public. HP2 described how *“pools close a lot because there’s nobody there that will open the pool. You need your lifeguard there for the pool to be open and they’re not there*”. Whilst many women expressed interest in sport, particularly football, basketball and softball, no women were currently involved in any sport with many stating this was due to *“no one to organise it”* (W5).

Participants noted walking as a means of exercise as Western concepts mainly done by ‘Balandas’ (Europeans) that might not be appropriate in this context. W3 described how “*some people shame”* when it comes to exercising, which was further iterated by W1 who described how there seems to “*be a bit of embarrassment about walking*.” Women spoke of programs such as walking groups that have been tried in the past that were not sustained due to ‘*lots of people coming and going’* C1. Women reiterated that successful programs must understand the specific needs and workings of that community. “*If they’re not from community, they might be told where to go*” (W1). Partaking in physical activity, purely to improve health, did not factor as a priority for participants, but instead, as highlighted by W6, physical activity was usually thought of either as incidental “*walking around. Going to visit other families”*, or in the context of a community activity to be enjoyed: “*look, they love their sport. It’s just that we don’t have it. And I think it’s time that that needs to change”* (HP3). Getting women together for organised sports was viewed positively and women suggested using facilities at the Youth Centre, Women’s Centre and recreation halls for these events. It was stressed that it needed to be culturally appropriate, with most women wanting exercise for women only, “s*omewhere in the private area, private place, not open place*” (W11).

#### Competing priorities

Engaging in lifestyle changes did not factor highly in the day-to-day lives of participants. Many women highlighted their role as a mother and prioritising the needs of their family as reasons for this. One health professional explains:

*“they are looking after their family. There is no me, it’s we. So, they are doing things for other people… Women are often trying to survive rather than focus on health and long-term outcomes*” (HP5).

It was often asserted that other life stressors would take priority because “y*ou’re trying to get through that day, you don’t care how you get your food or whatever it is, you just get something*” (HP2).

### Enablers to lifestyle change

#### Culture and connection with Country

Enablers to improved lifestyle were women’s connections to the land and cultural identity. Women described a healthier lifestyle when they were on Country; with improved diet when eating bush tucker. W7 reports *“at outstation, out bush we have good food but here [in community] we sometimes I drink coke*.” Women also talked about doing more incidental exercise on Country: “*people they get exercise when they go out hunting too*” (W1).

 The ‘community’ culture, where participants thought of themselves in terms of their relationship with other people and their community was a clear theme and an enabler for women to partake in group activities. Unlike more ‘individualistic’ cultures, women were not concerned as to who would look after their children whilst they partook in a lifestyle program, *“I’d bring them along, there’s lots of family*” (W7). It was also an enabler to partake in group sport. There was a strong feeling that the community knew what they wanted, and any successful programs needed to be community driven. HP1 asserted that “*the community’s got the answers. They’ve got the ideas and that’s what you need and that’s where all this funding needs to go*”.

### Solutions

#### Structural change

Some women thought that making access to unhealthy food and drink options more difficult would help people make healthier choices. W4 thought we should “*take it [soft drink] off the shelf*” and W3 suggested *“maybe lower the prices [of healthy foods].”* Others thought making unhealthy choices more expensive would result in people spending a higher proportion of their money on these options. W1 stated: *“people would still buy it. They buy alcohol and smokes, so…*” Several women stated that healthy food options were currently available in the stores and stores were already subsidising and promoting healthy food choices:

*“It would also be good if the shop put an emphasis on the healthy food. I’ve noticed these shop keepers have moved… the fruit and veg used to be down the back in a fridge. They’ve moved it to the front door”* (HP6).

#### Cooking classes and community kitchen

Potential programs such as a community kitchen or cooking classes were perceived well but needed to fit within the social structure of the community. Women generally thought a community kitchen would be a good idea: “*she was show us, showing us how to cook, how to make good food for the little ones and for ourself.”* (W10). Participants thought many women didn’t know how to cook, as described by C1: *“they need to learn that, cooking class….try to tell them … they can cook, in a place they can cook like a safe house.”* It was emphasised that cooking classes need to be culturally appropriate, as W7 suggested “*Bush tuckers and Balanda. Yeah, you know come together and… maybe they’ll get all the womens together and sit and talk about what sort of food that we want to, you know, eat, some better food. And bush tuckers…*” Some health professionals were concerned that “*the people that come to the education sessions that we have, we’re preaching to the converted. We’re not reaching the ones that really need it”* (HP2). They thought we needed to think of innovative ways to reach those who needed such programs the most: *“it’s not the script we need for our Indigenous communities. They need a different … The whole thing needs to be revamped*” (HP1).

#### Metformin for prevention of diabetes

Four of the 11 women reported they would take metformin to prevent diabetes after pregnancy and 2 of the 7 health professionals reported they would recommend this. Health professionals thought metformin was medicalising what is broadly a social issue: *“that’s a load of rubbish [giving metformin for prevention]. It’s just us again throwing a tablet at someone*” (HP1). When given the option of choosing a lifestyle change program or taking metformin preventatively, 8 of 11 women and 5 of 7 health professionals chose a lifestyle program.

## Discussion

In this study, we engaged health providers and Aboriginal women with the aim of exploring their views on a postpartum program. We report four key findings. Firstly, individual and community level programs must occur in parallel with structural changes designed to address food insecurity and adequate facilities to exercise. Secondly, programs need to be community driven and co-designed with participants. Thirdly, programs should be grounded in Aboriginal conceptions of health and finally, lifestyle programs were preferred over metformin.

Community lifestyle interventions should occur in parallel with policies at a government level. Like other remote Aboriginal communities, community A and B have experienced a recent history of colonisation with enduring effects, including substandard housing, food insecurity, psycho-social stressors and a lack of economic and occupational opportunities [31]. Although traditional food practises continue to play an important role within community, colonisation brought rapid lifestyle change with adverse changes in physical activity and nutrition [32, 33]. In our study, food insecurity was a key issue repeatedly identified by participants as a barrier to eating well. Gorton et al’s review on interventions to address food insecurity in high-income countries, reported a lack of evidence that community programs alone effectively reduce food insecurity [34]. Instead, governmental efforts to improve household incomes and reduce overcrowding, alongside community programs, would likely go further to reduce food insecurity [34].

There is substantial evidence for the benefits of healthy diet and physical activity in preventing and improving type 2 diabetes and cardiovascular disease [5]. However, despite the high rates of these conditions among Indigenous populations, examples of lifestyle programs are rare. Two systematic reviews on physical activity in Indigenous populations in North America [35] and in Australia and New Zealand [36] report both a small number of interventions and a lack of evaluations of these interventions. Hence, it is currently unclear how successful programs are, at increasing activity levels for Indigenous populations worldwide, and certainly there is no evidence for programs in the postpartum period.

What is clear, and consistent with our findings, is that programs initiated by the community are more likely to be sustained and have positive health effects [15, 37, 38]. A systematic review of nutrition programs for Aboriginal Australians report the most important factor determining the success of such programs is community control of development and implementation [15]. The Healthy Communities Project was a multi-component strategy that partnered with community leaders to reduce sugary drink consumption in remote communities in Queensland. Engagement from a range of stakeholder groups resulted in increased water sales and decreased sugary drink sales [39]. A community driven diabetes prevention program in a Maori rural community in New Zealand had similar positive results, with the investigators attributing its success to community ownership [40]. Our research, with participants reporting a desire for programs to be community driven, adds to the body of literature supporting the co-design of lifestyle programs. Co-design is considered best practice in research involving Indigenous peoples, and defined as meaningful end-user engagement in research design with engagement across all stages of the research process, with clear guidelines in New Zealand, Australia and Canada [41–43]. As evidenced by a diabetes prevention program for Aboriginal people in Western Australia, direction from community was vital for ensuring relevant application of previous research and effectiveness [44]. .

Connection to and appreciation of Country holds great importance to many Aboriginal People [45]. As reported by Thompson et al., being 'on Country' plays an important economic, dietary and cultural role in many communities [45] and we found participants expressed improved physical and mental health when they felt connected to Country and expressed a desire for dietary programs to incorporate traditional food practices. Similarly, in a study among Cree women with a history of GDM, the importance of incorporating traditional elements into a diabetes prevention program was underscored [46]. A systematic review on the benefits associated with engagement in Aboriginal Land Management activities (Australia and Canada), reported such engagement to be associated with significant health benefits through improvements in diet, activity, autonomy, and social and spiritual connection to land [47]. Like exercise programs, rather than approaching diet through the biomedical model, Aboriginal peoples’ understandings of food systems and well-being should provide the foundation from which to reset the narrative in relation to diet and health [48].

One’s primary role as a family and community member was evident throughout our interviews. As described in the literature [37], many of our participants expressed shame if participating in exercise for personal reasons rather than for the benefit of the community or family. Whilst the concept of shame has been variously described, in Aboriginal culture it can refer to situations in which a person is singled out, or the centre of attention and the associated concern regarding what others would think [49]. Shame is reportedly one of the largest hindrances to young Aboriginal women participating in physical activity [50]. Hence, individual style programs such as walking groups are unlikely to work in this context. Generally, women in our study were not interested in joining such groups due to (i) the individual nature and associated shame and (ii) the reliance of previous programs on people outside of the community to run them and the transient nature of such people. Alternatively, group sports, that give women a sense of collective identity from being part of a team, are more likely to be effective and could aid in keeping women connected to family and community [51]. In addition, women expressed a desire for group sport to be for women only.

Given the known barriers to lifestyle change, we asked participants for their perspective on using metformin to prevent diabetes. Metformin was not preferred by most of our participants. There were concerns with adherence given its gastrointestinal side effects and large size and practical considerations around length of treatment. Furthermore, all participants were able to identify clear gaps in lifestyle optimisation and expressed a preference to address these gaps prior to medicalising what many considered to be an environmental and structural issue.

Strengths of this study include the extensive involvement of (i) local Aboriginal Community Workers who were crucial in establishing a culturally safe space and ensuring appropriate interpretation of Aboriginal women’s voices and (ii) the guidance of the ATSIAG, who were actively involved throughout the project. There were several limitations. Generalizability and replicability of our findings are limited. Indigenous women are diverse, and any new program needs to be adapted to meet the specific needs of women and their cultural context. We were unable to return to communities to discuss themes in person with Aboriginal Community Workers due to COVID-19 travel restrictions. However, to cross-check the interpretation of findings and minimise biases, AW and the Community Worker spoke after each interview and SG analysed all transcripts. There was a smaller number of women recruited from community B compared to community A due to travel restrictions; however, themes were consistent across the two communities.

## Conclusions

This research concludes that programs should be community driven and grounded in Aboriginal conceptions of health. The Diabetes across the Lifecourse Northern Australia Partnership plans to co-design a program with Aboriginal and Torres Strait Islander women and communities to support healthy lifestyle before, during and after pregnancy. We will use experience-based co-design, to engage, gather experiences, understand, and determine priorities and then implement and evaluate the co-designed programs [52]. To ensure the development of culturally appropriate, effective programs, further studies need to be implemented and evaluated with Aboriginal people. In practice, this should occur alongside structural policies designed to address food insecurity and disparities in the social determinants of health, as experienced by Aboriginal people in remote communities.

## Supplementary information

**Additional file 1**

## Data Availability

Data are available from the authors upon reasonable request and with permission of the Human Research Ethics Committee of the NT Department of Health and Menzies School of Health Research.

## Abbreviations

GDM: Gestational Diabetes
NT: The Northern Territory
ATSIAG: Aboriginal and Torres Strait Islander Advisory Group
