# Supplementary material for: Incorporating Aboriginal women’s voices in improving care and reducing risk for women with diabetes in pregnancy - A phenomenological study
Source: BMC Pregnancy Childbirth. 2021 Sep 16;21:624. doi: 10.1186/s12884-021-04055-2 (PMC8445012; doi:10.1186/s12884-021-04055-2)
Supplement: Supplementary file 1 — Additional file 1 [file 12884_2021_4055_MOESM1_ESM.docx]

**Supplementary file**

**TITLE PAGE**

**Title: Incorporating Aboriginal women’s voices in improving care and reducing risk for women with diabetes in pregnancy - A phenomenological study.**

Anna J Wood^1^, Sian Graham^1,2^, Jacqueline A Boyle^1,3^, Beverley Marcusson-Rababi^1^, Shonada Anderson^1^, Christine Connors ^4^, Harold D McIntyre^5^, Louise Maple-Brown^1^, Renae Kirkham^1^

*1. Menzies School of Health Research, Charles Darwin University, NT, Australia*

*2. Indigenous Reference Group, Menzies School of Health Research, Charles Darwin*

*3. Monash Centre for Health Research and Implementation, Monash University, Vic, Australia*

*4. Top End Health Service, Northern Territory Department of Health, NT, Australia*

*5. Mater Research, The University of Queensland, South Brisbane, QLD, Australia*

**Semi-Structured Interview Guide- Women with a history of gestational diabetes (GDM)**

1. **General questions**
   1. What do you know about type 2 diabetes?
      1. How /why do people get type 2 diabetes?
   2. Is there anything you can do to stop this from happening?
   3. Do you think it is difficult to eat healthily and exercise after you have had a baby?
      1. What makes it difficult/easier?
2. **Dietary program**

This section is about what you eat and your weight

*(Background questions)*

- 1. Do you think you could eat better? And if yes-
     1. What is stopping you from improving what you eat?
  2. Do you or anyone at your home (or in your family) cook?
     1. What do you/they cook and how often?
     2. Do you prefer to buy hot food from the shop or food you have to prepare? Why?
     3. Why do you think a lot of people buy take away food rather than cooking?

*(Education and implementation of a dietary program)*

Would you like to learn more about eating healthily?

What types of things would you like to learn about?

Have you ever been to or seen a cooking class?

1. If yes- can you tell me more about this?
2. If no- do you think cooking classes could be a good idea?

What sorts of things would you like to learn how to cook/make?

*(Access to healthy food)*

1. Can you get healthy food from your store?
2. Do many people in your community buy these foods?
3. How could we help people to buy more healthy food and eat more healthy food?
4. **Exercise program**

This section is about exercise

*(Background questions)*

- 1. Do you do any exercise like walking or playing sport?
     1. If yes-what type of exercise?
  2. What makes exercise easy/hard?
  3. Would you like to exercise more?
  4. What sort of exercise do people in your community like to do?

*(Implementation of an exercise program)*

1. Do any of your friends or family exercise?

Have you ever been in an exercise group like a walking group?

1. If yes: how did this happen/who ran it/what was it like/would you like to do something like this again?

Would you be interested in a group exercise program?

Would you be interested in any of the following?

1. Aerobics, Walking group, Swimming, Group sport

*If yes to any of the above- what stops you from doing them now?*

*(Access to exercise facilities)*

1. Are there any places you can exercise?
2. How could we help people to exercise more?

*(Feedback on exercise)*

1. Do you think a health coach or someone to motivate you to exercise would help?
2. **Metformin**

This section is about the medication metformin

*(Background questions)*

- 1. Were you on any medications for your diabetes when you were pregnant?
     1. If yes which ones?

Have you heard of metformin? (if not on metformin when pregnant)

If yes; what have you heard

If you were on metformin when you were pregnant:

- - 1. Did you take it?
    2. Did you have any side effects, if so what were they?
       1. Did it make you feel sick, if so in what ways?

*(Taking metformin to prevent type 2 diabetes)*

To try and prevent women developing gestational diabetes again in their next pregnancy and to prevent type 2 diabetes, we could consider giving metformin to women after they were pregnant.

1. If you were feeling well, do you think it would be hard to take tablets every day? Why/why not?
2. Would you take a tablet twice a day, every day for a long time to prevent getting type 2 diabetes?
3. One of the main side effects people complain of is that it can make you feel sick.
   - 1. Have you heard this? And if so, what have you heard?
     2. On the one hand metformin might prevent diabetes but on the other hand it can make you feel sick, like you want to vomit and can give you diarrhoea, knowing this- do you think you would take this?
4. We think metformin is safe in breastfeeding and we often recommend it for women with type 2 diabetes who are breastfeeding, but we do know that it is in the breast milk. Would this put you off taking it?

How could we share information to women and their families about why this medication is important?

**Conclusion**

So we have talked about different ways me might prevent diabetes- about what you eat, exercise and taking metformin. Do you think you would be interested in any of these options?

What do you think you would be most interested in?

What would you really like to see in community/ is there something you think would benefit you?

**Semi-Structured Interview Guide- Women with a history of type 2 diabetes**

1. **General questions**
   1. What do you know about type 2 diabetes?
      1. How /why do people get type 2 diabetes?
   2. Is there anything you can do to stop this from happening?
   3. Do you think it is difficult to eat healthily and exercise after you have had a baby?
      1. What makes it difficult/easier?
2. **Dietary program**

This section is about what you eat and your weight

*(Background questions)*

- 1. Do you think you could eat better? And if yes-
     1. What is stopping you from improving what you eat?
  2. Do you or anyone at your home (or in your family) cook?
     1. What do you/they cook and how often?
     2. Do you prefer to buy hot food from the shop or food you have to prepare? Why?
     3. Why do you think a lot of people buy take away food rather than cooking?

*(Education and implementation of a dietary program)*

Would you like to learn more about eating healthily?

What types of things would you like to learn about?

Have you ever been to or seen a cooking class?

1. If yes- can you tell me more about this?
2. If no- do you think cooking classes could be a good idea?

What sorts of things would you like to learn how to cook/make?

*(Access to healthy food)*

1. Can you get healthy food from your store?
2. Do many people in your community buy these foods?
3. How could we help people to buy more healthy food and eat more healthy food?
4. **Exercise program**

This section is about exercise

*(Background questions)*

- 1. Do you do any exercise like walking or playing sport?
     1. If yes-what type of exercise?
  2. What makes exercise easy/hard?
  3. Would you like to exercise more?
  4. What sort of exercise do people in your community like to do?

*(Implementation of an exercise program)*

1. Do any of your friends or family exercise?

Have you ever been in an exercise group like a walking group?

1. If yes: how did this happen/who ran it/what was it like/would you like to do something like this again?

Would you be interested in a group exercise program?

Would you be interested in any of the following?

1. Aerobics, Walking group, Swimming, Group sport

*If yes to any of the above- what stops you from doing them now?*

*(Access to exercise facilities)*

1. Are there any places you can exercise?
2. How could we help people to exercise more?

*(Feedback on exercise)*

1. Do you think a health coach or someone to motivate you to exercise would help?
2. **Metformin**

This section is about the medication metformin

*(Background questions)*

1. Are you taking medication for your diabetes?
2. If yes which ones?

*(If you are on metformin):*

1. Do you take it?
2. Do you have any side effects?
3. Did you breastfeed your baby? Did you take metformin whilst breastfeeding?
4. We think metformin is safe in breastfeeding and we often recommend it for women with type 2 diabetes who are breastfeeding, but we do know that it is in the breast milk. Would this put you off taking it?
   - 1. Do you think this might be a worry for other women? Why?

*(If you are not on metformin)*

1. Have you ever taken medications? Do you find it hard?
2. If you were feeling well, do you think it would be hard to take tablets every day? Why/why not?
3. Metformin is a big tablet; do you think you would take this every day to prevent type 2 diabetes?
4. One of the main side effects people complain of is that it can make you feel sick.
5. Have you heard this? And if so, what have you heard?
6. On the one hand metformin can lower your sugar levels but it can make you feel sick, like you want to vomit and can give you diarrhoea, knowing this- would you take it?
7. We think metformin is safe in breastfeeding, but we do know that it is in the breast milk. Would this put you off taking it?
8. Do you think this might be a worry for other women? Why?
9. How could we share information to women and their families about why this medication is important?

**Conclusion**

So, we have talked about different ways me might prevent diabetes- about what you eat, exercise and taking metformin. Do you think you would be interested in any of these options?

What do you think you would be most interested in?

What would you really like to see in community/ is there something you think would benefit you?

**Semi-Structured Interview Guide- Health Professionals**

1. **General questions**
   1. Do you think women with GDM or T2DM during pregnancy know that they can improve outcomes with lifestyle changes?
   2. What do you think are the main barriers to women making lifestyle changes postpartum?
   3. What would make it easier?
   4. Who do you think they should hear messages from and why?
2. **Dietary programme**

This section is about what women eat and their weight

*(Background questions)*

1. Generally speaking, do you think women in your community could eat better?
2. What do you think is stopping them from improving what they eat?
3. Did you think women know that weight loss could prevent and improve type 2 diabetes?

*(Education and implementation of a dietary programme)*

Do you think education is an issue i.e. do women know about how to eat healthily?

Do you think something like cooking classes could help?

1. If yes- can you tell me more about this? -

*(Access to healthy food)*

1. Do you think access to healthy food is an issue?
2. Do you think women and families have enough food available?
3. Can you get healthy food from your store?
4. What sort of things?
5. Do many people in the community buy these foods?
6. How could we help people to buy more healthy food?

1. **Exercise programme**

This section is about how much women exercise

*(Background questions)*

Do you think women in your community exercise much?

1. What type of exercise?

What do you think is preventing women from exercising more- particularly in the postpartum period?

Do you think women understand that exercising could prevent and improve diabetes?

*(Implementation of an exercise programme)*

1. Do you think education on the benefits of exercise is an issue?
2. Is there any type of exercise programme you think might work?

Do you think any of the following could work?

1. Aerobics
2. Walking group
3. Swimming
4. Resistance training/ cross fit
5. Group sport

*(Access to exercise facilities)*

1. Are there any places women can exercise in your community?
2. How could we help people to exercise more?
3. **Metformin**
   1. Do you think women who had GDM would take metformin postpartum to prevent getting type 2 diabetes?
      1. If not then why not?
   2. Do you think women with type 2 diabetes who are prescribed metformin take it?
      1. If not then why not?
   3. Do you feel comfortable with women taking metformin whilst breastfeeding?
